# Progression of Fibrinogen Decrease during High Dose Tigecycline Therapy in Critically Ill Patients: A Retrospective Analysis

**DOI:** 10.3390/jcm10204702

**Published:** 2021-10-13

**Authors:** Benedikt Treml, Sasa Rajsic, Tobias Hell, Dietmar Fries, Mirjam Bachler

**Affiliations:** 1 General and Surgical Intensive Care Unit, Department of Anaesthesiology and Critical Care Medicine, Medical University Innsbruck, 6020 Innsbruck, Austria; benedikt.treml@tirol-kliniken.at (B.T.); dietmar.fries@tirol-kliniken.at (D.F.); mirjam.bachler@tirol-kliniken.at (M.B.); 2 Department of Mathematics, Faculty of Mathematics, Computer Science and Physics, University of Innsbruck, 6020 Innsbruck, Austria; tobias.hell@uibk.ac.at

**Keywords:** antibiotics, coagulation disorder, coagulopathy, glycylcycline, hypofibrinogenemia, infection, tigecycline, tygacil

## Abstract

Tigecycline is a novel glycylcycline broad-spectrum antibiotic offering good coverage for critically ill patients experiencing complicated infections. A known side effect is a coagulation disorder with distinct hypofibrinogenemia. To date, the information on possible risk factors and outcomes is sparse. Therefore, the aim of this study is to examine the time course of fibrinogen level changes during tigecycline therapy in critically ill patients. Moreover, we sought to identify risk factors for coagulopathy and to report on clinically important outcomes. We retrospectively reviewed all intensive care patients admitted to our General and Surgical Intensive Care Unit receiving tigecycline between 2010 and 2018. A total of 130 patients were stratified into two groups based on the extent of fibrinogen decrease. Patients with a greater fibrinogen decrease received a higher dose, a longer treatment and more dose changes of tigecycline, respectively. In regard to the underlying pathology, these patients showed higher inflammation markers as well as a slightly reduced liver synthesis capacity. We, therefore, conclude that such a fibrinogen decrease may be based upon further impairment of liver synthesis during severe inflammatory states. To decrease the risk of bleeding, cautious monitoring of coagulation in critically ill patients treated with high-dose tigecycline is warranted.

## 1. Introduction

Tigecycline is a novel glycylcycline broad-spectrum antibiotic. The glycylcyclines originate from tetracyclines with structural alterations making them suitable for broad-spectrum treatment of severe Gram-negative, Gram-positive, and anaerobic infections, including certain multi-drug-resistant strains [1]. They are primarily designed to overcome two main mechanisms of tetracycline resistance, either by the acquisition of new genes that code for efflux pumps of tetracycline or via a protein in charge for the protection of bacterial ribosomes from tetracycline action [2]. Currently, the main indications that could be addressed by tetracycline analogues are complicated intra-abdominal infections, complicated skin and skin-structure infections, community-acquired bacterial pneumonia and other infections caused by vancomycin-resistant Enterococcus (VRE) or methicillin-resistant Staphylococcus aureus (MRSA) [3]. All of these are seen regularly at intensive care units (ICU) [4].

Critically ill patients often suffer from complicated medical or surgical conditions, exposing them to the development of multi-drug-resistant infections, leading to longer hospital stays, higher mortality and increased costs [5,6,7,8,9].

A known and described side effect of tigecycline therapy is a coagulation disorder, reported with a low incidence [10,11]. In the European Medicines Agency product information sheet, a “laboratory measurement of decreased ability to form blood clots” is recognized as a possible side effect, in addition to a low platelet count and hypofibrinogenemia (which may affect one in a thousand patients), which are recognized as uncommon and rare side effects, respectively [10]. However, reports of glycylcycline-related coagulopathy are increasing constantly [9].

The current literature on coagulopathy related to tigecycline use is still limited [9]. Until 2019, only four small scale studies [12,13,14,15] with 134 patients, 13 case reports and one review of available literature existed [9]. In a Chinese retrospective analysis, Leng et al. demonstrated decreased fibrinogen levels in 50 hospitalized patients [14]. In 2020 and 2021, four further retrospective studies with more than 600 patients were published (three from China and one from Spain), showing a possible association of tigecycline use with hypofibrinogenemia most commonly commencing on the sixth day of treatment [16,17,18,19]. In a retrospective study from China, 50.5% of 426 analyzed patients developed hypofibrinogenemia, which appeared to be more frequent in patients with kidney failure [16]. Two studies of 45 and 20 ICU patients reported decreased fibrinogen levels during high-dose glycylcycline treatment [12,13] and one study found an association between the duration of treatment and coagulopathy [20]. Active bleeding, requiring treatment with plasma and/or fibrinogen, was reported in 20% of patients [13]. Other than these sparse data, eight case reports regarding either coagulopathy [21,22,23,24] or severe and life-threatening coagulation disorders exist [25,26,27,28].

However, the main reason for a decrease in fibrinogen during glycylcycline treatment or an occurrence of coagulopathy afterwards remains unclear [9]. Furthermore, this drug reaction could be related to an increased consumption or a hepatic synthesis problem. To date, a few studies with a rather small number of ICU patients are available in the literature and the information on possible risk factors and outcomes is sparse. Therefore, the aim of this study is to examine the course of fibrinogen level changes during tigecycline therapy of critically ill patients, to provide more detail on potential risk factors and predictors of coagulation disorder and to report on clinically important outcomes.

## 2. Materials and Methods

### 2.1. Patient Selection

We retrospectively reviewed the electronic medical charts of all patients admitted to the General and Surgical ICU between 1 January 2010 and 31 December 2018. This tertiary ICU of the Department of Anaesthesiology and Critical Care Medicine within the Medical University of Innsbruck, Austria, treats surgical, polytraumatized, and medical patients.

All critically ill patients undergoing tigecycline treatment (Tygacil^®^ 50 mg powder for intravenous infusion, anatomical therapeutic chemical code (ATC) J01AA12, Pfizer Europe MA EEIG, Belgium; approval number EU/1/06/336/001) were enrolled. Exclusion crtieria included patients younger than 18 years, cases of treatment with either a duration of less than three days or two independent tigecycline administrations with less than nine days between them (corresponding to five half-life times of 42 h), commencement of therapy before admission to our university hospital, and patients with incomplete data sets.

Patients were stratified into two groups based on the highest proportional decrease in fibrinogen. Dosing was based upon clinical judgement of the treating physicians and resulted in high-dose regimes with an intravenous loading dose of at least 100 mg. Moreover, the duration of treatment as well as the changing of dosage depended upon severity and location of the infection.

### 2.2. Data Collection

We obtained: (1) socio-demographic data including age, sex, body weight, height and body mass index; (2) underlying infection, antibiotic treatment within ten days before tigecycline initiation and pathogens resistance; (3) date and time of first tigecycline application, duration of treatment and the date of therapy termination; (4) amount of tigecycline daily dosage (mg/d), total received amount of tigecycline and potential change of standard dosage; (5) coagulation status including platelets count (g/L), fibrinogen Clauss (mg/dL), prothrombin time (PT, %), activated partial thromboplastin time (aPTT, seconds) and antithrombin (%); (6) other laboratory parameters as hemoglobin (g/L), hematocrit (L/L), erythrocytes (T/L), white blood cells (WBC, g/L), protein (g/dL), C-reactive protein (CRP, mg/dL), creatinine (mg/dL), bilirubin (mg/dL), serum glutamic oxaloacetic transaminase (u/L) and serum glutamic pyruvic transaminase (u/L); and finally (7), data on ICU mortality and in-hospital mortality.

Data were recorded starting seven days before the begin of tigecycline treatment, on the day of the first application and daily during the whole treatment period until the end.

Two authors (B.T., S.R.) independently checked each electronic medical chart and extracted the data in the predesigned case report form.

This retrospective study was approved by the Ethics Committee of the Medical University of Innsbruck, Austria (#1084/2019).

### 2.3. Statistical Analyses

A mathematician not involved in the study procedures or patient assessment performed the statistical analyses using R, version 3.4.2 (free software for statistical computing and graphics—R Core Team 2014: a language and environment for statistical computing. R Foundation for Statistical Computing, Vienna, Austria). All statistical assessments were two-sided and a significance level of 5% was used. The Wilcoxon rank sum test and Fisher’s exact test were applied to assess differences between two groups. We present continuous data as medians (25th–75th percentile) and binary variables as no./total no. (%). We show effect size and precision with estimated median differences for continuous data and odds ratios (OR) for binary variables, with 95% Confidence Intervals (CI).

#### 2.3.1. Mean over Rolling Time

To compensate for daily fluctuations of lab parameters, the mean over rolling time windows of three consecutive days was applied. Supplemental Figure S1 shows the agreement of the means of all trajectories (blue) and means of corresponding rolling-window-mean trajectories (red) with all single rolling-window-mean trajectories in the background.

#### 2.3.2. Stratification

Based on our clinical observations, we defined a decrease in fibrinogen of more than 25% from the initial value as being clinically significant. We therefore separated all patients into two groups, based on the highest proportional decrease in fibrinogen in the mean over rolling time windows of three consecutive days of more than 25%. All patients with a fibrinogen decrease of more than 25% were grouped into the fibrinogen-decrease group.

#### 2.3.3. Regression Model

A generalized additive model (GAM) based on a Gaussian distribution was fit in order to analyze the relationship between a selected response variable (e.g., fibrinogen in the first model, variable name: “fib”) and the two regressor variables “time” and “dec”, which indicates whether the observation belongs to a patient with or without fibrinogen decrease. The time variable was included via a smoothing function which allows for high flexibility in modeling the time effect.

Hence, the first model where fibrinogen depends on the smooth function of time and the logical variable “dec” was set up with the formula:

fib ~ dec + s(time).

The regression model showed an estimated mean difference in a decrease in fibrinogen levels of 70.55 mg/dL (56.86 to 84.23, *p* < 0.0001).

## 3. Results

Over a period of nine years, 3089 patients were admitted to our ICU. After screening all their medical charts, 181 patients met the inclusion criteria, with 130 patients showing complete data sets. The demographics of the patients are displayed in Table 1. Patients were predominantly male and slightly overweight upon admission.

### 3.1. Underlying Diseases and Prior Antibiotic Treatment

Table 1 depicts the underlying infections and indications for tigecycline treatment. Interestingly, 86.2% of all microbiological findings showed a normal susceptibility, 6.2% revealed highly resistant Gram-positive and 7.7% highly resistant Gram-negative pathogens. The most frequent microorganism was Staphylococcus, followed by Enteroccoccus (Table S1). The majority of patients received, within ten days of tigecycline treatment initiation, penicillins and cephalosporins, followed by carbapenemes (Table S2).

### 3.2. Fibrinogen Course

**Figure 1:**
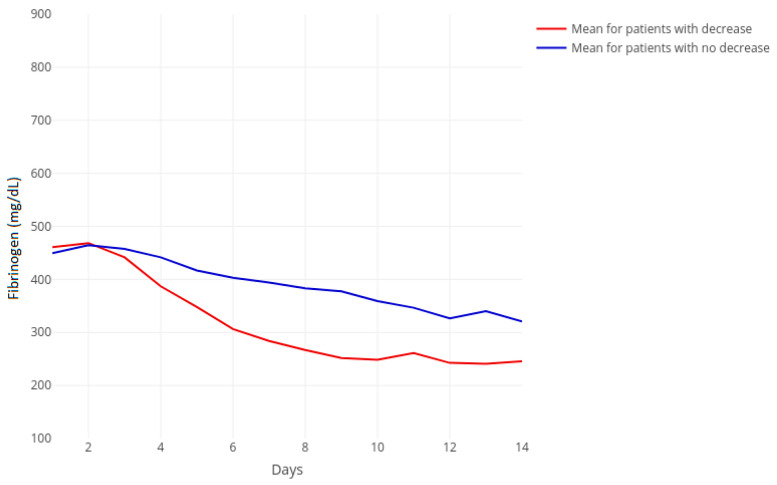
Time course of fibrinogen separated for decreasing (red, *n* = 59) and non-decreasing (blue, *n* = 71) groups with corresponding groupwise mean. The horizontal axis represents days of treatment beginning at zero as the timepoint seven days before treatment. Day *n* represents the *n*th day of treatment.

Seven days before and at the start of tigecycline therapy, fibrinogen levels were comparable in both groups. Interestingly, two days after treatment initiation, fibrinogen started to decrease in both groups with a steeper decrease in the decreased fibrinogen group that was statistically significant after seven days. After ten days of treatment, fibrinogen levels in the fibrinogen-decrease group remained low, around values of 300 mg/dL, compared to the higher fibrinogen in the no-fibrinogen-decrease group (Figure 1).

### 3.3. Standard Coagulation Parameters and Platelets Course

Before treatment, PT and aPTT were comparable in both groups. However, the fibrinogen-decrease group experienced a greater increase in PT and aPTT than the no-fibrinogen-decrease group.

Antithrombin levels were lower in the fibrinogen-decrease group before the start of tigecycline. Thereafter, antithrombin decreased in both groups, maintaining the difference between both groups throughout the course of treatment.

Platelets were comparable before the start of treatment and showed a contrasting course after a few days, with a slight decrease in the fibrinogen-decrease group and an increase in the no-fibrinogen-decrease group.

### 3.4. Biomarkers of Inflammation Course

**Figure 2:**
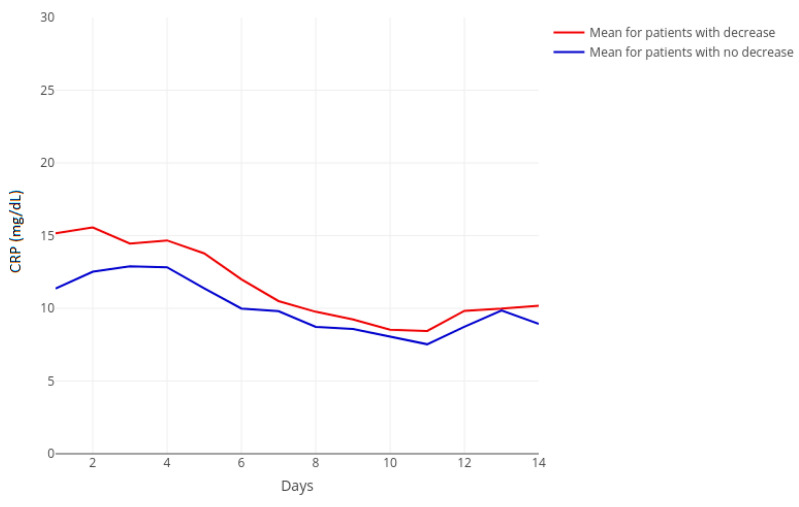
Temporal course of C-reactive protein (CRP) separated for decreasing (red, *n* = 59) and non-decreasing (blue, *n* = 71) groups with corresponding groupwise means. CRP reflects C-reactive protein. The horizontal axis represents days of treatment beginning at zero as the timepoint seven days before treatment. Day *n* represents the *n*th day of treatment.

Before tigecycline treatment started, CRP levels were higher in the fibrinogen-decrease group. During tigecycline treatment, CRP decreased in both groups until twelve days of treatment, with a slight increase thereafter (Figure 2).

**Figure 3:**
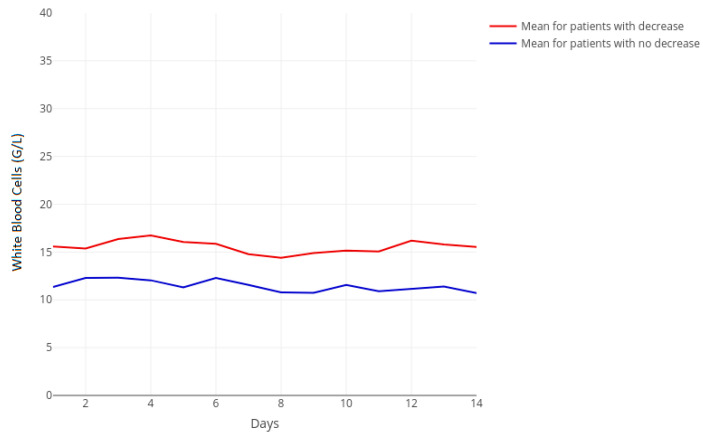
Temporal course of white blood cell (WBC) count separated for decreasing (red, *n* = 59) and non-decreasing (blue, *n* = 71) groups with corresponding groupwise means. WBC reflects white blood cell count. The horizontal axis represents days of treatment beginning at zero as the timepoint seven days before treatment. Day *n* represents the n^th^ day of treatment.

White blood cell (WBC) counts showed distinctly higher values before treatment started, especially in the fibrinogen-decrease group. Interestingly, the course of WBCs remained unchanged during the whole tigecycline treatment period (Figure 3).

### 3.5. Organ Function Parameters Course

Creatinine, serum glutamic oxaloacetic transaminase (GOT), serum glutamic pyruvic transaminase (GPT) and protein remained unchanged. Bilirubin was slightly higher in the no-fibrinogen-decrease group.

### 3.6. Other Parameters Course

Erythrocytes, hemoglobin and hematocrit remained unchanged during the whole observation period.

## 4. Discussion

In this retrospective single-center study, we demonstrated a decreasing fibrinogen course throughout high-dose treatment with tigecycline in 130 critically ill patients. Moreover, patients with a greater fibrinogen decrease received longer treatments and higher doses of tigecycline and experienced more dose changes. Moreover, CRP levels and WBC counts were higher at the beginning, which is in line with the work of Campany-Herrero et al., who identified initial CRP levels of more than 25 mg/dL being a risk factor for hypofibrinogenemia in 62 patients [26].

### 4.1. Fibrinogen Decreases during High-Dose Tigecycline Therapy

Patients in the fibrinogen-decrease group received a higher total amount of tigecycline and a higher dosage per kilogram. This is comparable with Hu and co-workers [18] reporting a dose higher than 100 mg/day being a risk factor in 127 critically ill patients. All studies defined a daily dose of 200 mg as high-dose as compared to the recommended standard dose of 2 × 50 mg/day [12,13,14,16,17,18,19]. Intriguingly, our patients received doses that were about one-third lower than in the other studies (153 mg/day in the decreasing group, 130.4 mg/day in the non-decreasing group), but experienced comparable fibrinogen decreases, which raises the question of whether or not fibrinogen decrease is dose-dependent. In contrast to this, Liu et al. only observed a trend towards higher doses in 15 patients with low fibrinogen [19]. Furthermore, the largest trial did not show an association of fibrinogen decrease with higher doses, but instead with treatment durations longer than 14 days [16].

The weights of patients are rarely reported in the available literature. Only one study reported weights of 20 patients with peritoneal carcinomatosis receiving a calculated daily dose of 1.37 mg/kg/die [15]. This is in contrast to our overweight patients receiving an approximately one-third higher dosage with a similarly comparable fibrinogen decrease. The most recent study from China only provided BMI [19], so the current literature still remains conflicted in regard to dose-dependency of coagulopathy.

Given the pharmacodynamic properties of tigecycline, a high-dose regimen (at least 2 × 100 mg/day) appears to be more effective than a lower one. This is even more relevant as tigecycline is often used as an antibiotic of second choice or last resort in critically ill patients. In our work, 14% of the patients showed highly resistant bacteria.

Cunha and co-workers first reported a high-dose tigecycline therapy being effective in treating serious systemic infections [29]. Moreover, several trials showed high doses of tigecycline being associated with better clinical and microbiological outcomes as compared to a standard dose [30,31,32,33,34]. This is in contrast to a retrospective work in 134 ICU patients with ventilator associated pneumonia (VAP) showing a comparable 28-day mortality with low and a high doses of tigecycline [35].

### 4.2. Fibrinogen Decreases during Long-Term Tigecycline Treatment

The decreasing group experienced a longer tigecycline treatment by two days, which is in line with the findings of Liu et al. [19]. Three recent studies in critically ill patients showed similar results, with Hu et al. observing a treatment duration nearly twice as long (11 vs. 6 days) if fibrinogen decreased below 200 mg/dL [18]. Chinese [16] and Spanish research [17] showed a treatment of longer than two and four weeks, respectively, being a risk factor as well.

### 4.3. Inflammatory Burden, Organ Function and Fibrinogen Decrease

The question arises whether or not the fibrinogen decrease per se is attributable to the high levels of inflammation, as this can impair liver synthesis function [35,36]. This could explain the more distinct fibrinogen decrease in the decreasing group with higher WBC counts and lower antithrombin levels. However, CRP decreased in both groups, converging towards the end. Moreover, recent data showed comparable inflammatory markers in critically ill patients with and without fibrinogen decrease before tigecycline treatment [18].

Consistent with a more impaired hepatic synthesis in the fibrinogen-decrease group, the lower antithrombin levels, lower PT and a prolonged aPTT are in line with previous works [12,14,15].

Despite scant liver synthesis impairment, we could not observe tangible alterations to renal or global hepatic organ function, which corresponds to recent findings using either low or high doses of tigecycline [13,14,17,18,19,21]. However, only one study showed renal failure as being a risk factor for tigecycline-induced hypofibrinogenemia [16]. With one-third of tigecycline being eliminated by urine excretion, renal impairment might not cause drug accumulation or an increased toxicity [37,38].

Lastly, a high inflammatory burden can explain a minor decrease in platelets after seven days in the fibrinogen-decrease group. Such a sepsis-induced thrombopenia [39,40] is in line with Leng et al. [14]; however, in contrast to this, the largest trial in critically ill patients found unchanged platelets [16].

### 4.4. Mortality

We found no difference in mortality between both groups, with an ICU mortality of 31%. This is nearly twice as high as our expected ICU mortality of around 15% (data not shown), which clearly reflects the severity of (inflammatory) disease in patients treated with tigecycline. Our hospital mortality is comparable with a Greek study (36%) [12], but lower than the one from China (more than 50%) [18]. Furthermore, we observed a trend towards an increased mortality in the fibrinogen-decrease group without reaching significance. This is in line with Liu et al. [19], who reported a higher hospital mortality in patients with hypofibrinogenemia compared to those with normal fibrinogen (41.1% vs. 25.9%). However, these patients were not critically ill.

### 4.5. Mechanism of Coagulopathy

The key underlying reason for coagulopathy following glycylcycline treatment remains unclear. The decreasing group experienced either: 1) an increased consumption of fibrinogen, or 2) an impaired hepatic fibrinogen synthesis as part of the severe systemic inflammation. Fibrinogen has an essential function in blood clotting, cellular and matrix interactions, inflammation, fibrinolysis, angiogenesis and even in neoplastic processes [41]. Hypofibrinogenemia in general may occur due to impaired fibrinogen synthesis, assembly, intracellular processing or domain secretion [42]. In critically ill patients with high levels of inflammation, low fibrinogen levels may reflect ongoing low-grade consumption and deposition or development of disseminated intravascular coagulopathy [43,44].

The current literature provides data primarily on coagulopathy symptoms with only a few tenuous explanations of the potential mechanisms. Antibiotic treatment is often associated with coagulation disorders, by reducing intestinal microflora and affecting vitamin K_2_ (menaquinone) production [45,46,47], but not influencing the synthesis of fibrinogen. Furthermore, the inhibition of cytokines due to tigecycline interaction could be one of the reasons for an impaired or even reduced fibrinogen production [23]. Tigecycline was supposed to impede interleukin six (IL-6) expression, which normally stimulates gene expression and increases fibrinogen blood levels [48,49]. Recently, a case report showed a tigecycline-induced inhibition of mitochondrial DNA translation with possible mitochondrial dysfunction [50]. From a clinical point of view, it is crucial to know if the administration of fibrinogen concentrate could further fuel the underlying mechanism in cases of increased consumption.

Our group showed qualitative changes in the architecture of the fibrin network with increasing doses of tigecycline in vitro, without observing an effect on clot stability. Therefore, peripheral interactions of tigecycline on fibrin polymerization are not the reason for such coagulopathy [51]. None of the previously mentioned mechanisms can completely explain the coagulopathy related to the tigecycline use, and further studies could still shed light on this issue.

### 4.6. Limitations

This study is limited in several aspects. Due to the retrospective nature of this study, selection bias cannot be excluded. The plasma concentration of fibrinogen may have been confounded by circumstances not covered by this work. We are neither able to establish nor dismiss a causal relationship between fibrinogen decrease and the observed laboratory values change, as all of the critically ill patients received other drugs concomitantly. However, as all of our patients experienced a distinct decrease in fibrinogen, the chance of a significantly confounding factor in only one of the groups should be rather small. Additionally, minor bleeding events during tigecycline therapy may have been overlooked. Another limitation is the criteria applied for patient stratification, which is based on our own clinical observations. Lastly, larger samples of patients are needed for further research.

## 5. Conclusions

To the best of our knowledge, this is the largest European study investigating hypofibrinogenemia during tigecycline therapy, a still poorly understood side effect. We examined the trajectories of the fibrinogen decrease and other coagulation parameters as well as organ function parameters in 130 critically ill patients. Moreover, we report weight-adjusted doses for the first time. Based on our findings, we recommend stricter dose adjustment based on the weight of patient, cautious monitoring of fibrinogen and coagulation parameters in severely inflamed patients receiving high-dose tigecycline.

## Figures and Tables

**Table 1:** Patients’ characteristics, overview of tigecycline treatment and mortality ^a^.

	Total (*n* = 130)	No Fibrinogen Decrease (*n* = 71)	Fibrinogen Decrease (*n* = 59)	Estimate with 95% CI ^b^	*p* Value ^c^
Demographic data					
Age	67 (59–75)	70 (58–78)	68 (59–73)	−1 (−5 to 2)	0.5304
Female sex	42 (32.3%)	23 (32.4%)	19 (32.2%)	0.99 (0.44 to 2.21)	1
Weight (kg)	80 (68–90)	80 (70–99.5)	75 (62.5–87.5)	9 (2 to 16)	0.0133
Height (m)	1.72 (1.63–1.8)	1.72 (1.62–1.78)	1.72 (1.65–1.8)	0 (−0.04 to 0.03)	0.8022
BMI (kg/m^2^)	26.04 (23.46–30.77)	27.46 (24.09–33.03)	25.17 (21.91–28.34)	2.66 (0.54 to 4.83)	0.0142
Underlying infection					
Community-acquired bacterial pneumonia	27 (20.8%)	17 (23.9%)	10 (16.9%)	0.65 (0.27 to 1.55)	0.3865
Intra-abdominal infection	45 (34.6%)	21 (29.6%)	24 (40.7%)	1.63 (0.79 to 3.38)	
Skin or skin-structure infection	18 (13.8%)	12 (16.9)	6 (10.2%)	0.56 (0.20 to 1.59)	
Proven or suspected highly resistant pathogen	40 (30.8)	21 (29.6%)	19 (32.2%)	1.13 (0.54 to 2.39)	
Tigecycline treatment					
Days of Tigecycline treatment	10 (8–14)	10 (6–13)	12 (8–14)	−2 (−4 to 0)	0.0229
Tigecycline dosage (mg/day/kg)	1.82 (1.43–2.22)	1.63 (1.33–1.91)	2.04 (1.74–2.4)	−0.4 (−0.6 to −0.21)	0.0002
Total amount of Tigecycline (mg/kg)	18.34 (11.23–27.27)	14.09 (9.15–23.48)	22.97 (15.56–32)	−7.47 (−11.11 to −3.85)	0.0001
Change of Tigecycline dosage	26/127 (20.5%)	9/69 (13%)	17/58 (29.3%)	2.74 (1.04 to 7.71)	0.0283
Mortality					
Death during ICU stay	40 (31%)	18 (25.7%)	22 (37.3%)	1.71 (0.76 to 3.91)	0.1834
Death during hospitalization	45 (36.9%)	22 (32.4%)	23 (42.6%)	1.55 (0.69 to 3.48)	0.2626

^a^ Binary data are presented as no./total no. (%), continuous data as medians (25th to 75th percentile); ^b^ odds ratios for binary variables and estimated median difference for continuous variables; ^c^ assessed by Fisher’s Exact Test for categorical variables and Wilcoxon Rank Sum Test for continuous variables; CI, Confidence Intervals; ICU, intensive care unit; BMI, body mass index.

## Data Availability

The data sets used and analyzed during the current study are available from the corresponding author on reasonable request.

## Supplementary Materials

The following are available online at https://www.mdpi.com/article/10.3390/jcm10204702/s1, Figure S1: Comparing mean of all trajectories (blue) and mean of corresponding rolling-window-mean trajectories (red) with all single rolling-window-mean trajectories in the background, Table S1: Type of pathogen and pathogen resistance identified in patient samples, Table S2: Class of antibiotics used in the treatment of patients within ten days before Tigecycline initiation.
