# Supplementary material for: Progression of Fibrinogen Decrease during High Dose Tigecycline Therapy in Critically Ill Patients: A Retrospective Analysis
Source: J Clin Med. 2021 Oct 13;10(20):4702. doi: 10.3390/jcm10204702 (PMC8537220; doi:10.3390/jcm10204702)
Supplement: Supplementary file 1 [file jcm-10-04702-s001.zip › jcm-1404433-supplementary.pdf]

Supplementary Materials

Table S1. Type of pathogen and pathogen resistance identified in patient samples[a].

	**All patients**	**No fibrinogen decrease**	**Fibrinogen decrease**	***p* value [b]**
Type of pathogen	127 (100%)	69 (54.3%)	58 (45.7%)	0.1660
Empirical therapy	44 (34.6%)	24 (34.8%)	20 (34.5%)	
Staphylococcus	25 (19.7%)	14 (20.3%)	11 (19.0%)	
Enterococcus faecalis/faecium	22 (17.3%)	14 (20.3%)	8 (13.8%)	
Stenotrophomonas maltophilia	12 (9.4%)	6 (8.7%)	6 (10.3%)	
Klebsiella	10 (7.9%)	3 (4.3%)	7 (12.1%)	
Escherichia coli	6 (4.7%)	5 (7.2%)	1 (1.7%)	
Enterobacter	4 (3.1%)	3 (4.3%)	1 (1.7%)	
Other[c]	4 (3.1%)	0 (0%)	4 (6.9%)	
Pathogen resistance	130 (100%)	71 (54.6%)	59 (45.4%)	0.2779
Normal susceptibility	112 (86.2%)	64 (90.1%)	48 (81.4%)	
Highly resistent gram-positive pathogen	8 (6.2%)	4 (5.6%)	4 (6.8%)	
Highly resistant gram-negative pathogen	10 (7.7%)	3 (4.2%)	7 (11.9%)	

[a] Binary data are presented as no./total no. (%); [b] Assessed by Fisher's Exact Test; [c] Other (Serratia, Burkholderia cepacia, Acinetobacter, Citrobacter).

Table S2. Class of antibiotics used in the treatment of patients within ten days before Tigecycline initiation[a].

	**All patients**	**No fibrinogen decrease**	**Fibrinogen decrease**	***p* value [b]**
Penicillins and Cephalosporins	83 (63.8%)[c]	42 (59.2%)	41 (69.5%)	0.2219
Carbapenems	79 (60.8%)	41 (57.7%)	38 (64.4%)	0.4387
Glycopeptides	58 (44.6%)	30 (42.3%)	28 (47.5%)	0.5523
Fluoroquinolones	41 (31.5%)	23 (32.4%)	18 (30.5%)	0.8177
Oxazolidinones	29 (22.3%)	16 (22.5%)	13 (22%)	0.9455
Fosfomycin	19 (14.6%)	13 (18.3%)	6 (10.2%)	0.1908
Nitroimidazoles	16 (12.3%)	12 (16.9%)	4 (6.8%)	0.0803
Tetracycline	14 (10.8%)	7 (9.9%)	7 (11.9%)	0.7134
Lincosamides	12 (9.2%)	8 (11.3%)	4 (6.8%)	0.3787
Cotrimoxazole	11 (8.5%)	7 (9.9%)	4 (6.8%)	0.5299
Macrolides	10 (7.7%)	4 (5.6%)	6 (10.2%)	0.3339
Polymyxins	9 (6.9%)	7 (9.9%)	2 (3.4%)	0.1479
Rifamycines	8 (6.2%)	4 (5.6%)	4 (6.8%)	0.7866
Aminoglycosides	7 (5.4%)	5 (7.0%)	2 (3.4%)	0.3583
Lipopeptides	3 (2.3%)	3 (4.2%)	0 (0%)	0.1101

[a] Binary data are presented as no./total no. (%); [b] Assessed by Fisher's Exact Test; [c] Values showing number (%) of patients receiving the drug.

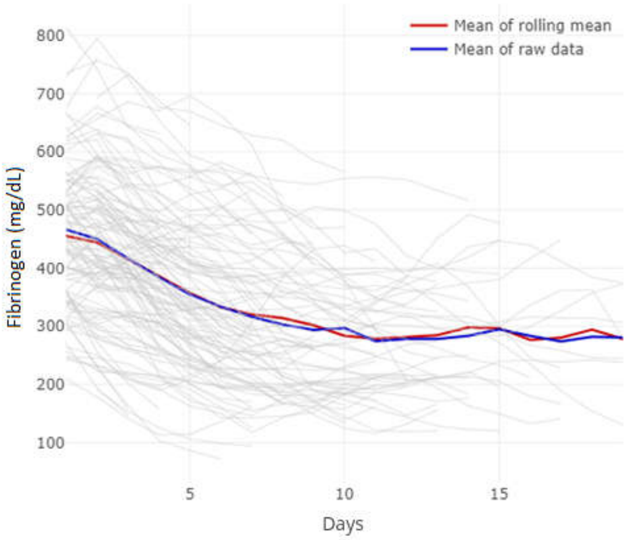

Figure S1. Comparing mean of all trajectories (blue) and mean of corresponding rolling-window-mean trajectories (red) with all single rolling-window-mean trajectories in the background.
